# Tenosynovitis

**Published:** 1882-09

**Authors:** William Barton Hopkins


TENOSYNOVITIS; ITS CAUSES, NATURE, SYMPTOMS AND TREATMENT: BASED UPON AN ANALYSIS OF FIFTEEN CASES. By WILLIAM BARTON HOPKINS, M.D., etc. [Read June 7, 1882.]

TENOSYNOVITIS may be defined as an affection usually occurring in the forearm, and characterized by a peculiar creaking of the tendons as they move in their sheaths, depending upon a particular kind of strain to which the muscles belonging to these tendons have been subjected.

*Cause.*—The predisposing cause of the affection is the occupation of the individual, and in studying, therefore, fifteen cases, occurring in subjects of otherwise average health, the nature of their employment is worthy of special attention. In three of the fifteen, the disease occurred in men employed in a dye-house, whose work consisted in wringing the goods, which had been soaked in dye; in two, the patients were weavers, who throw the shuttle from side to side with the index finger of the right hand; one case occurred in a baker, from kneading bread; one in a boiler riveter, from hammering; one in a car driver, from using the brake; one in an iron moulder, from continued use of the shovel; one in a plaster worker, from stirring plaster with a hoe; one in a washerwoman, from using a clothes wringer; one in a laborer, who continued to work after receiving a severe contusion of the forearm from the fall of a heavy iron pipe; and one each in a rope twister, a marble rubber, and a painter.

In contrasting the above-named occupations with many others, requiring far more muscular effort, and giving employment to many more workmen than these, the idea suggests itself, that it is not the mere amount of strain to which the muscles and their tendons are put, that predisposes to the disease, but rather the kind of effort, which is of a tedious, continuous, monotonous sort. On the other hand, trades which would appear likely to furnish subjects for the disease more frequently than those which have been already spoken of, fail to do so. This, in some instances, can be explained. Gold beating, for example, where an eight-pound hammer is used almost uninterruptedly for five hours, and is carried from above the shoulder down to the level of the waist, would seem to contradict this view, as the disease is unknown to one of the largest gold leaf manufacturers; a careful study of the movements of the operatives in performing this work, however, shows that the strain is not upon the muscles of the forearm, but rather upon those of the shoulder and arm; as the hammer descends simply by gravity and returns by recoil from the elastic block, composed of alternate sheets of gold and animal membrane, to a point where the biceps and deltoid muscles complete the elevation.

The exciting cause of the attack is usually the resumption of work to which the individual is thoroughly accustomed, after a shorter or longer interval, when he is out of practice, and when the parts involved in executing special movements have become less actively nourished; though in the case of the washerwoman, the clothes wringer was used for the first time, and the rope twister was doing work that was new to him. In the laborer the attack was of traumatic origin.

*Pathology.*—The means of determining the exact lesion in this disease are necessarily to a certain extent conjectural, but as the pain and crepitation are coincident in their onset and subsidence, as there is no impairment of motion after recovery has occurred, and, as the parts under treatment regain their normal condition in a very short time, it seems highly probable that there is no true inflammatory process at all, certainly none extending beyond the stage of congestion, and that the creaking which exists is due to insufficient lubrication, with consequent drynesss, not, as has been supposed, to exudation of lymph. Under rest and counter-irritation the congestion very soon disappears, the synovial surfaces pour out their proper fluid, and the tendons once more move smoothly and noislessly in their sheaths.

*Symptoms.*—Soreness, amounting to positive pain upon motion or pressure along the course of the affected tendons, inability to use the part, and the presence of the peculiar creaking, which is communicated to the finger on palpation, are the symptoms which denote the existence of tenosynovitis.

*Diagnosis.*—From its common seat upon the dorsum of the forearm, this affection may be mistaken for fracture of the radius. The history of the case, however, showing that there has been no blow or fall, as a rule ; the quality of the crepitus, which is much softer and finer than that of fracture, and like that of cellular emphysema after fracture of the ribs, or that produced by rubbing two pieces of cloth between the fingers, and the way in which the crepitation may be elicited,—all leave little chance of error. The disease will not be mistaken for a strain of the muscle, if a careful physical examination is made.

*Treatment.*—From what has been already said, it will be seen that the disease is at once acute, painful, and disabling. It, however, yields, as a rule, readily to treatment ; for the patient can seldom work more than a day after he is attacked, and finding that he exhausts the usual home embrocations, without relief, promptly seeks aid elswhere ; this enables the surgeon to institute treatment before an advanced stage is reached and permament mischief done by a disposition of plastic matter. Absolute rest of all the parts concerned is the most important element in the treatment ; a palmar splint, therefore, from the elbow to the tips of the fingers is applied, when the forearm is the part affected. Counter-irritation is next indicated, and may be employed in one of two ways. If the skin is red, a band one inch broad of tincture of iodine should be painted in an oval form around the area over which creaking is felt ; while a lotion of lead water and laudanum is applied within this band. In cases where there is but slight creaking, and no redness of the skin, tincture of iodine may be painted directly over the diseased part, without the employment of any lotion. The dressing is re-applied each day, until all pain, tenderness, and creaking have disappeared, which generally occurs at the end of four or five days. After this a roller bandage alone is continued, until the parts have regained their tone.—*Phil. Medical Times.*

